# NK Cells Regulate CD8^+^ T Cell Mediated Autoimmunity

**DOI:** 10.3389/fcimb.2020.00036

**Published:** 2020-02-13

**Authors:** Philipp A. Lang, Sarah Q. Crome, Haifeng C. Xu, Karl S. Lang, Laurence Chapatte, Elissa K. Deenick, Melanie Grusdat, Aleksandra A. Pandyra, Vitaly I. Pozdeev, Ruifeng Wang, Tobias A. W. Holderried, Harvey Cantor, Andreas Diefenbach, Alisha R. Elford, David R. McIlwain, Mike Recher, Dieter Häussinger, Tak W. Mak, Pamela S. Ohashi

**Affiliations:** ^1^Princess Margaret Cancer Centre, Campell Family Institute for Breast Cancer Research, University Health Network (UHN), Toronto, ON, Canada; ^2^Department of Gastroenterology, Hepatology and Infectious Diseases, University of Düsseldorf, Düsseldorf, Germany; ^3^Department of Molecular Medicine II, Medical Faculty, Heinrich Heine University Düsseldorf, Düsseldorf, Germany; ^4^Department of Immunology, University of Toronto, Toronto, ON, Canada; ^5^Toronto General Hospital Research Institute and UHN Transplant, University Health Network, Toronto, ON, Canada; ^6^Institute of Immunology, University Hospital Essen, University of Duisburg-Essen, Essen, Germany; ^7^Immunology Division, Garvan Institute of Medical Research, Darlinghurst, NSW, Australia; ^8^Faculty of Medicine, UNSW Sydney, Sydney, NSW, Australia; ^9^Department of Hematology, Oncology and Rheumatology, University Hospital Bonn, Bonn, Germany; ^10^Department of Cancer Immunology and Virology, Dana-Farber Cancer Institute and Department of Immunology, Harvard Medical School, Boston, MA, United States; ^11^Laboratory of Innate Immunity, Department of Microbiology, Infectious Diseases and Immunology, Charité - Universitätsmedizin Berlin, Berlin, Germany; ^12^Berlin Institute of Health (BIH), Berlin, Germany; ^13^Mucosal and Developmental Immunology, Deutsches Rheuma-Forschungszentrum, Berlin, Germany; ^14^Medical Outpatient Clinic and Immunodeficiency Lab, University Hospital Basel, Basel, Switzerland

**Keywords:** CTL, LCMV, IFN-α, autoimmunity, pathology

## Abstract

Elucidating key factors that regulate immune-mediated pathology *in vivo* is critical for developing improved strategies to treat autoimmune disease and cancer. NK cells can exhibit regulatory functions against CD8^+^ T cells following viral infection. Here we show that while low doses of lymphocytic choriomeningitis virus (LCMV-WE) can readily induce strong CD8^+^ T cell responses and diabetes in mice expressing the LCMV glycoprotein on β-islet cells (RIP-GP mice), hyperglycemia does not occur after infection with higher doses of LCMV. High-dose LCMV infection induced an impaired CD8^+^ T cell response, which coincided with increased NK cell activity during early time points following infection. Notably, we observed increased NKp46 expression on NK cells during infection with higher doses, which resulted in an NK cell dependent suppression of T cells. Accordingly, depletion with antibodies specific for NK1.1 as well as NKp46 deficiency (*Ncr1*^*gfp*/*gfp*^ mice) could restore CD8^+^ T cell immunity and permitted the induction of diabetes even following infection of RIP-GP mice with high-dose LCMV. Therefore, we identify conditions where innate lymphoid cells can play a regulatory role and interfere with CD8^+^ T cell mediated tissue specific pathology using an NKp46 dependent mechanism.

## Introduction

For decades, efforts have been made to understand ways to promote the induction of CD8^+^ T cell immunity as an avenue to improve tumor immune therapy, promote viral clearance, or treat autoimmune diseases. Many factors are known to influence T cell activation and function, such as the maturation status of antigen-presenting cells (APC) the expression of co-inhibitory or co-stimulatory molecules and the cytokine microenvironment. Although immunotherapeutic strategies targeting co-inhibitory molecules have revolutionized cancer treatment (Pardoll, 2012; Ribas and Wolchok, 2018), it is clear that additional work is required to better define the criteria for strong immune responses *in vivo* in order to further develop and/or refine existing immunotherapies.

Various immune cell populations, such as regulatory T cells have been shown to impact CD8^+^ T cell responses (Mempel et al., 2006). Studies have also demonstrated that innate lymphoid cells including NK1.1^+^ cells in mice or CD56^+^ cells in humans have displayed immune-regulatory functions and can play an important role in limiting CD8^+^ T cell responses (Crome et al., 2013). ILCs/NK cells regulate CD8^+^ T cell anti-viral immunity (Su et al., 2001; Lu et al., 2007; Lang et al., 2012; Waggoner et al., 2012), and CD8^+^ T cell antitumor immunity (Iyori et al., 2011; Iraolagoitia et al., 2016; Crome et al., 2017; Picard et al., 2019).

NK cell activity is orchestrated by a wide variety of activating and inhibiting receptors on NK cells. For example, elevated NKG2D-Ligand expression on activated T cells may trigger their susceptibility to NK cell regulation, presumably by binding to NKG2D activating receptors on NK cells (Rabinovich et al., 2003; Lang et al., 2012). Furthermore, type I interferon (IFN-I) can suppress expression of ligands for the activating NK cell receptor NKp46 (Crouse et al., 2014). Hence, IFN-I is important to protect anti-viral T cells against NK cell mediated attack (Crouse et al., 2014; Xu et al., 2014). IFN-I can induce expression of MHC-I and MHC-Ib molecules, such as Qa-1b, which bind to inhibitory NK cell receptors and reduce NK cell mediated regulation of anti-viral T cells (Xu et al., 2014, 2017). Furthermore, lack of the inhibitory NK cell receptor, 2B4, is associated with increased NK regulatory activity and limited T cell immunity during infection (Waggoner et al., 2010). Moreover, NK cells may target CD4^+^ T cells for killing and thus prevent T cell help to cytotoxic T cells (Waggoner et al., 2012).

We have used the RIP-GP model to dissect the events that are required for the activation of CD8^+^ effector T cell function that is sufficient to induce tissue destruction *in vivo*. In this model, transgenic expression of the lymphocytic choriomeningitis virus glycoprotein (LCMV-GP) is restricted to the β-islet cells of the pancreas via the rat insulin promotor (RIP). In this system, CD8^+^ T cell mediated destruction of the islets can be induced by LCMV infection, which lead to hyperglycemia and diabetes in these animals. Similarly, mature dendritic cells pulsed with LCMV-GP peptides can also trigger the expansion of tissue specific LCMV-GP reactive T cells, which recognize and destroy β-islet cells expressing LCMV-GP (Ohashi et al., 1991; Dissanayake et al., 2011; Lin et al., 2011). Furthermore, IFN-I increases MHC-I expression on β-islet cells during infection, which is critical for T cell infiltration into the pancreas and diabetes progression (Lang et al., 2005, 2009).

In the present study, we found that RIP-GP mice infected with a high dose of LCMV showed a modest reduction in CD8^+^ T cell immune responses and did not develop diabetes. High dose, but not low dose LCMV infection resulted in rapid NK cell activation and expression of NKp46 after infection. Accordingly, depletion of NK cells or NKp46 deficiency in mice restored CD8^+^ T cell immunity and induction of autoimmunity in animals infected with high doses of LCMV. These studies demonstrate that NK cells can significantly limit CD8^+^ T cell responses by NKp46 *in vivo* and have a profound impact on tissue destruction.

## Results

### Infection With a High Dose of LCMV Limits the Induction of Diabetes

**Figure 1:**
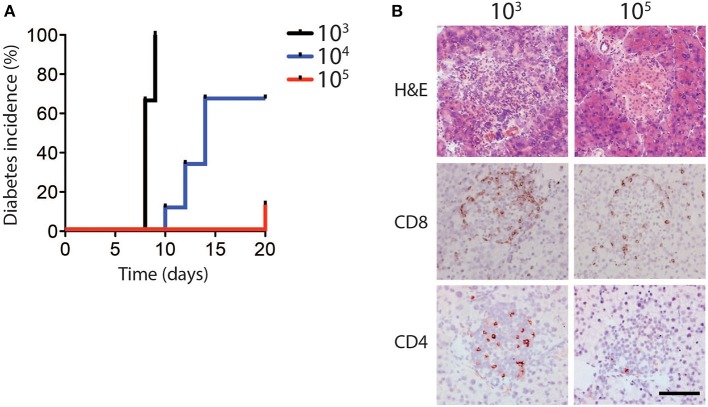
Diabetes incidence depends on dose of LCMV infection. **(A,B)** RIP-GP mice were infected with 10^3^, 10^4^, or 10^5^ PFU of LCMV WE. **(A)** Percent incidence of diabetes following LCMV infection at indicated doses was determined by glycaemia measurement (*n* = 8–9). **(B)** Immunohistochemistry of sections from snap frozen pancreas samples harvested from RIP-GP mice infected with 10^3^ and 10^5^ PFU of LCMV showing H&E, anti-CD8, and anti-CD4 staining. One representative image of *n* = 3–5 is shown (scale bar = 50 μm).

In order to determine whether different infectious doses of LCMV can influence the induction of CD8^+^ T cell mediated immune pathology, RIP-GP mice were infected intravenously with the LCMV WE strain ranging from 10^3^ to 10^5^ plaque-forming units (PFU) per mouse. Consistent with previous observations, all mice infected with low dose LCMV (10^3^ PFU) developed hyperglycemia, and subsequently diabetes, after ~10 days (Ohashi et al., 1991, 1993). Surprisingly however, only a fraction of the animals infected with an intermediate dose (10^4^ PFU), and very few of the mice infected with a high dose of LCMV (10^5^ PFU) became diabetic (Figure 1A, Supplementary Figure 1A).

Previous work has shown that induction of diabetes in RIP-GP mice is dependent on CD8^+^ T cell infiltration and destruction of pancreatic islets (Ohashi et al., 1991). Consistent with glycemic measurement, the pancreatic islets of mice infected with 10^3^ PFU of LCMV exhibited strong CD8^+^ and CD4^+^ T cell infiltration, compared to the limited infiltration after 10^5^ PFU of LCMV (Figure 1B). MHC-I expression levels on islet cells were similar in animals infected with a high dose and low dose LCMV (Supplementary Figure 1B), indicating that limited CD8^+^ T cell infiltration and lack of diabetes induction in high dose infection was likely not due to differential islet MHC-I expression (Ohashi et al., 1993; Lang et al., 2005), but may be related to altered T cell function.

### Infection With High Dose LCMV Generates Limited Numbers of Virus-Specific CD8^+^ T Cells

**Figure 2:**
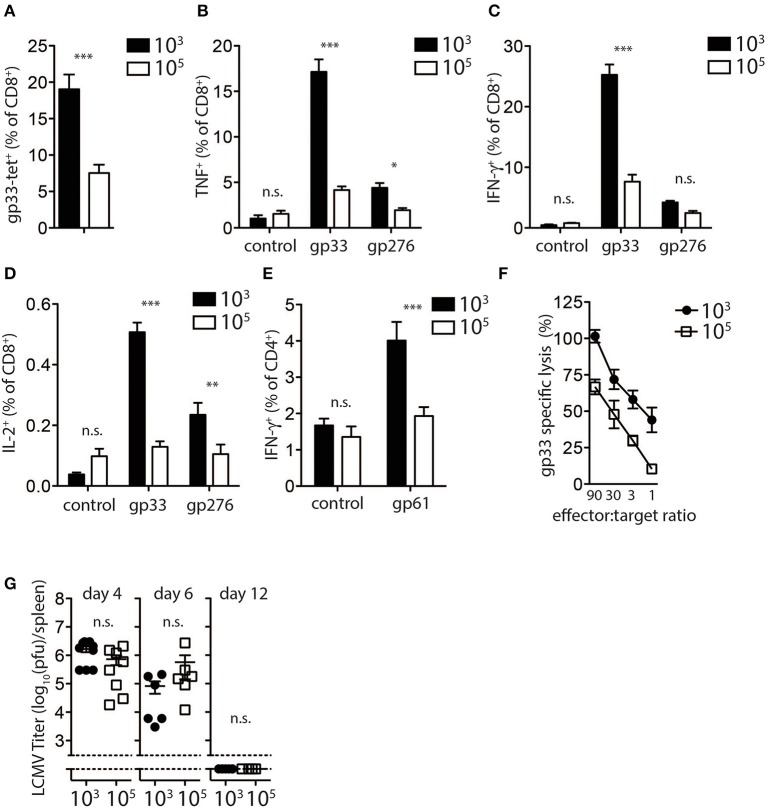
Expansion and function of virus specific CD8^+^ T cells depends on infectious dose of LCMV. **(A–E)** C57Bl/6 mice were infected with 10^3^ and 10^5^ PFU of LCMV-WE. T cells were analyzed on day 8 post-infection by flow cytometry or ^51^Cr release assay. **(A)** LCMV specific CD8^+^ T cells in blood samples were measured by tetramer staining. ***indicates *p* < 0.001, *n* = 8. **(B)** TNF-α production in CD8^+^ T cells after re-stimulation for 5 h with LCMV specific peptides determined by intracellular staining (***indicates *p* < 0.001, *indicates *p* < 0.05, *n* = 9–11). **(C)** Intracellular IFN-γ levels measured in CD8^+^ T cells of from single cell suspended splenocytes after re-stimulation with LCMV specific epitopes (***indicates *p* < 0.001, n.s. indicates not significant, *n* = 7–8). **(D)** Intracellular IL-2 levels measured in CD8^+^ T cells of from single cell suspended splenocytes after re-stimulation with LCMV specific epitopes (***indicates *p* < 0.001, **indicates *p* < 0.01, n.s. indicates not significant, *n* = 5). **(E)** IFN-γ production in CD4^+^ T cells after re-stimulation with the LCMV specific epitope gp61 measured by intracellular staining (***indicates *p* < 0.001; n.s. indicates not significant, *n* = 5). **(F)** Percent gp33 specific lysis determined by ^51^Cr release from labeled EL-4 target cells following incubation at the indicated splenocyte (effector)/target cell ratios (effector target ratio) (*n* = 6, left panel). **(G)** Virus titers were analyzed in spleen tissue at the indicated time points after LCMV infection by plaque assay (*n* = 3–6).

In order investigate the mechanism in which high dose LCMV impairs the induction of diabetes in this model, we analyzed CD8^+^ T cell responses following infection with different LCMV doses. LCMV-gp33 tetramer analysis in peripheral blood revealed that a significantly higher proportion of virus specific CD8^+^ T cells were present in the peripheral blood of low dose LCMV infected mice (Figure 2A). These effects were not restricted to the gp33 epitope as the percentage of CD8^+^ T cells recognizing the nucleoprotein-derived np396 epitope of LCMV was also increased following low dose infection (Supplementary Figure 2A). Next we evaluated the effect of LCMV dosage on the capacity of virus-specific CD8^+^ and CD4^+^ T cells to produce effector cytokines following *in vitro* LCMV-peptide re-stimulation. We found the proportion of TNF-α (Figure 2B), IFN-γ (Figure 2C), and IL-2 (Figure 2D) producing CD8^+^ T cells was 3–5-fold lower in high dose LCMV infected mice compared with mice infected with low dose LCMV (Figures 2B–D). Similar changes were observed for the T cells specific for the LCMV-nucleoprotein- derived epitope (Supplementary Figure 2B). Furthermore, the proportion of CD4^+^ T cells producing IFN-γ in response to the LCMV-derived immune-dominant MHC-II restricted helper cell epitope, GP-61, was also reduced in high dose vs. low dose infected mice (Figure 2E). Consistently, direct *ex-vivo* examination of T cell cytotoxicity revealed reduced cytotoxic killing capacity of T cells derived from mice infected with high-dose LCMV compared to T cells from mice inoculated with low dose LCMV (Figure 2F).

We next examined potential mechanisms to explain the observed reduced T cell activity and inability to induce diabetes following high dose LCMV infection. We examined the kinetics and the pattern of viral replication in mice infected with different doses of LCMV. Spleen viral titers at day 4, 6, and 12 post-infection were similar in low dose compared to high dose LCMV infected mice (Figure 2G). All mice had cleared LCMV by day 12 after infection from the spleen (Figure 2G). Thus, although induction of LCMV-specific CD8^+^ T cells was reduced in high dose LCMV infected mice and did not result in destruction of pancreatic islets *in vivo*, T cell immunity was still sufficient to potently eliminate the virus.

We next examined whether viral inoculum dose resulted in differential levels of LCMV in the pancreas. Replicating LCMV could not be measured in the pancreas at all-time points tested after either low or high dose LCMV infection (Supplementary Figure 3A). Immune-histologic staining for the LCMV nucleoprotein readily detected virus infected cells in the marginal zone of the spleen 3 days after low or high dose LCMV infection (Supplementary Figure 3B). However, infected cells could not be detected in the pancreas at any of the time points examined (Supplementary Figure 3C). Thus, we speculated that it is unlikely that the observed differences in tissue destruction in low dose vs. high dose LCMV infected mice are due to differential accumulation of LCMV antigen in the pancreas.

### NK Cells Limit the Generation of CD8^+^ T Cells Following High Dose LCMV Infection

**Figure 3:**
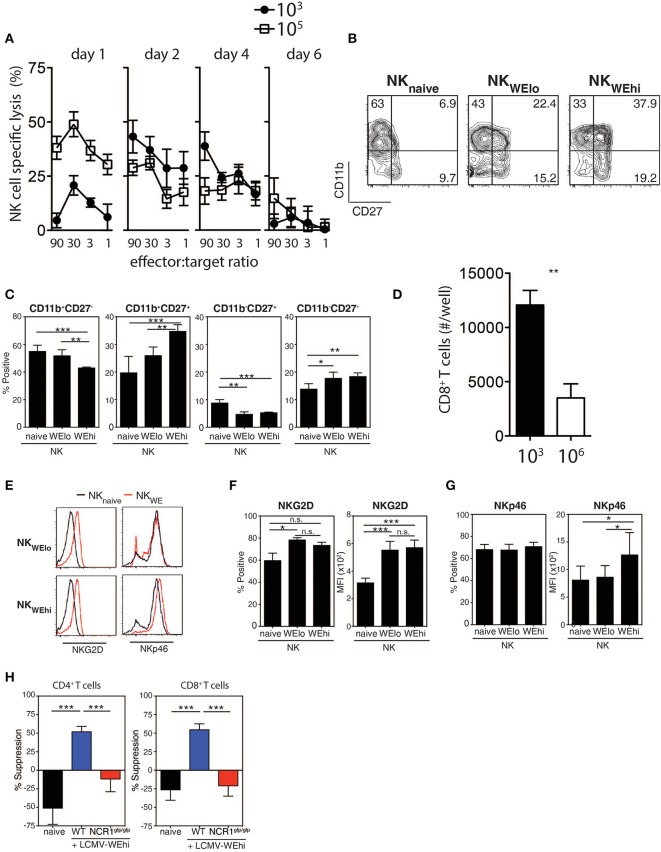
NK cells are activated during high dose LCMV infection. C57Bl/6 mice were infected with 10^3^ or 10^5^ PFU of LCMV WE at day 2 post-infection. **(A–C)** NK cell cytotoxicity was determined by ^51^Cr release assay on Yac-1 target cells (*n* = 4). **(B,C)** CD11b and CD27 expression were determined on NK cells (*n* = 7). **(D)** NK cells were harvested from animals infected with 10^3^ or 10^6^ PFU of LCMV. Following coincubation with T cells, CD8^+^ T cell number was assessed 48 h later (*n* = 3–5). **(E–G)** C57Bl/6 mice were infected with 10^3^ or 10^5^ PFU of LCMV WE. NKG2D and the NKp46 expression level was measured on NK cells from spleen tissue at day 2 post-infection. **(H)** NK cells were harvested from WT or NCR1^gfp/gfp^ animals infected with 10^6^ PFU of LCMV. Following coincubation with CD4^+^ T cells or CD8^+^ T cells, T cell suppression was determined (*n* = 4–13) (*indicates *p* < 0.05, **indicates *p* < 0.01, ***indicates *p* < 0.001).

Next, we examined whether the LCMV doses had an impact on the induction of NK cytotoxicity using YAC-1 target cells. NK cell mediated cytotoxicity was augmented early after high dose LCMV infection when compared with low dose infected animals (Figure 3A), which rapidly declined consistent with previous reports (Xu et al., 2017). Forty-eight hours after infection with LCMV high dose, NK cells exhibited higher expression of activation markers, such as CD11b, CD27, and 4-1BB when compared to low dose infected mice (Figures 3B,C, Supplementary Figure 4). To examine whether NK cells from high vs. low dose infected mice were able to directly kill CD8^+^ T cells, NK cells were harvested from infected animals and co-incubated with T cells *in vitro*. We observed reduced T cell numbers in the presence of NK cells obtained from 10^6^ PFU infected animals when compared to NK cells from 10^3^ PFU infected mice (Figure 3D). Next we examined potential mechanisms by which increased NK cell activity was observed after high dose infection. NK cells showed upregulation of the NK cell activating receptor NKG2D during LCMV infection (Figures 3E,F). However, the activating NK cell receptor NKp46 was selectively up-regulated following infection with high dose of LCMV in contrast to low dose infection (Figures 3E,G). Accordingly, NK cell mediated suppression of T cells was not detected when *Ncr1*^*gfp*/*gfp*^ NK cells were used compared to WT NK cells (Figure 3H). Therefore, NK cells from mice infected with high dose LCMV were able to kill T cells more readily than NK cells from low dose infected mice via an NKp46 dependent mechanism.

### NK Cells Prevent Diabetes Induction Following High Dose LCMV Infection

**Figure 4:**
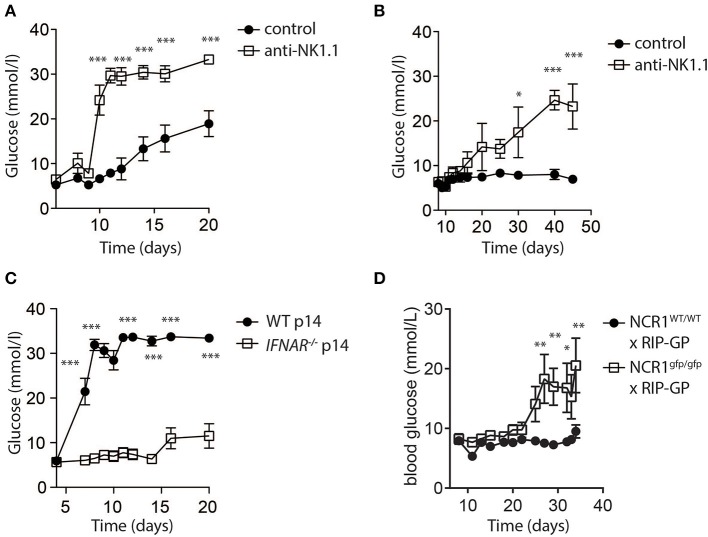
NK cells control CD8^+^ T cell induced autoimmune disease. **(A)** RIP-GP mice were infected with 10^5^ PFU of LCMV. One group (open squares) was treated with anti-NK1.1 on d-3 and d-1. Blood glucose levels were analyzed at indicated time points (*n* = 3–9, ***indicates *p* < 0.001; n.s. indicates not significant). **(B)** Mice were treated as in **(A)** but infected with 3x10^5^ PFU of LCMV (*n* = 3). **(C)** Equal amount of negatively sorted T cells from *P14*^+^ and *IFNAR*^−/−^*P14*^+^ mice were transferred into RIP-GP mice and infected with 10^5^ PFU of LCMV. Blood glucose levels were monitored at different time points (*n* = 6). **(D)** RIP-GP/NCR1^wt/wt^ and RIP-GP/NCR1^gfp/gfp^ were infected with 10^5^ pfu of LCMV. Blood glucose levels were monitored at different time points (*n* = 4) (*indicates *p* < 0.05, **indicates *p* < 0.01, ***indicates *p* < 0.001; n.s. indicates not significant).

While low dose LCMV infection induced autoimmunity in RIP-GP mice, high doses of LCMV failed to induce hyperglycemia (Figure 1A). In this model, evidence demonstrated that high dose LCMV infection activates NK cells to limit autoreactive CD8^+^ T cell responses. We next tested whether direct elimination of NK cells might be sufficient to alleviate the block in diabetes induction following high dose LCMV infection of RIP-GP mice. RIP-GP mice were treated with the NK-cell depleting antibody against NK1.1 and infected with high doses of LCMV-WE. All NK cell-depleted RIP-GP mice developed diabetes following high dose LCMV infection, whereas NK cell competent high dose LCMV infected RIP-GP mice remained euglycemic (≤14 mM) or showed significantly delayed hyperglycemia (Figures 4A,B). NK cell depletion did not change the initial virus replication in spleen or pancreas tissues nor the IFN-I production in the sera when compared to non-NK cell depleted control animals (Supplementary Figure 5).

We hypothesized that increased NK cell activity was triggered by the NK cell activating receptor NKp46. NKp46 ligands are suppressed on antigen specific T cells through IFN-I signaling, which renders IFNAR deficient T cells susceptible toward NK cell mediated attack (Crouse et al., 2014). Consistent with this hypothesis, when we adoptively transferred WT LCMV-GP specific T cells, the induction of diabetes in RIP-GP mice occurred following high dose infection due to increased antigen specific T cell immunity (Figure 4C). However, in absence of IFNAR on LCMV specific T cells, diabetes was not induced (Figure 4C) because the T cells express high levels of NKp46, leading to the activation of NK cells which in turn limits survival of LCMV-specific T cells. Accordingly, when we infected *Ncr1*^*gfp*/*gfp*^ X RIP-GP animals, the induction of diabetes was restored compared to WT RIP-GP controls, due to the lack of NKp46 expression which resulted in impaired induction of NK activity (Figure 4D). Taken together, these data indicate that NK cells inhibit the LCMV-gp specific CD8^+^ T cell response and prevent the induction of diabetes via the NK cell activating receptor NKp46.

### NK Cells Limit Anti-viral T Cell Immunity During High Dose Infection

**Figure 5:**
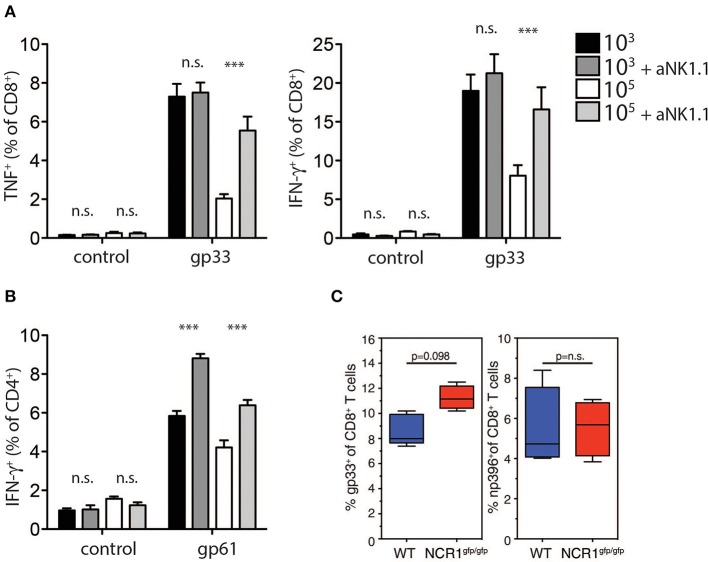
NK cell depletion restores T cell immunity in high dose infected animals. **(A,B)** RIP-GP and anti-NK1.1 treated RIP-GP mice were infected with 10^3^ or 10^5^ PFU of LCMV-WE **(A)** TNF-α production in CD8^+^ T cells after LCMV specific peptide restimulation for 5 h was determined by intracellular staining (left panel, ***indicates *p* < 0.001, *indicates *p* < 0.05, *n* = 6). Right panel: intracellular IFN-γ levels were measured in CD8^+^ T cells after restimulation with the indicated LCMV specific epitopes (***indicates *p* < 0.001, n.s. indicates not significant, *n* = 6) **(B)** IFN-γ production in CD4^+^ T cells after restimulation with the LCMV specific epitope gp61 was measured by intracellular staining (left panel, ***indicates *p* < 0.001; n.s. indicates not significant, *n* = 5). **(C)** RIP-GP × NCR1^wt/wt^ and RIP-GP × NCR1^gfp/gfp^ animals were infected with 10^5^ pfu of LCMV. Tetramer-gp33^+^ and tetramer-np396^+^ T cells were determined in spleen tissue (one of three independent experiments were shown *n* = 4–5).

Previous reports suggest that NK cell depletion improves T cell immunity during chronic viral infection (Lang et al., 2012; Waggoner et al., 2012). To investigate whether NK cells were responsible for the reduced generation and function of GP-specific CD8^+^ T cells in high dose LCMV infected mice, we depleted NK cells *in vivo* by administration of the depleting NK1.1 antibody before low or high dose LCMV infection. NK cell depletion rescued TNF-α and IFN-γ production in CD8^+^ T cells in mice infected with high dose LCMV, but did not alter CD8^+^ T cell function in low dose LCMV infected mice (Figure 5A). Notably, we observed a significant increase in CD4^+^ T cell mediated IFNγ production following NK cell depletion in mice infected with low and high doses of LCMV (Figure 5B). Moreover, we observed increased CD8^+^ T cell immunity following infection of NKp46 deficient mice with LCMV high dose when compared to WT animals (Figure 5C). Taken together we concluded that NK cells limit antigen specific T cell immunity and accordingly prevent establishment of autoimmune diabetes.

## Discussion

Here, we examined the effect of the infectious dose of LCMV on CD8^+^ T cell function and the induction of autoimmunity. Unexpectedly, RIP-GP mice infected with a high dose of LCMV showed limited induction of diabetes, whereas mice infected with low doses of virus developed diabetes within 2 weeks. The impaired induction of diabetes following high dose LCMV infection was associated with reduced numbers of circulating GP-specific CD8^+^ T cells, and correspondingly reduced cytokine production and cytotoxic function. Importantly, high dose LCMV infection provided the appropriate microenvironment that led to the induction of regulatory NK cells. This was demonstrated by the ability of NK cells from high dose infected mice to kill activated CD8^+^ T cells, while NK cells from low dose infected mice could not kill CD8^+^ T cells. Blocking this regulatory capacity of NK cells by either NK cell depletion *in vivo* or limiting the activation of NK cells using NKp46 deficient mice led to the induction of diabetes after infection with high dose virus. This demonstrates that NK cells regulate CD8^+^ T cell effector function *in vivo* against tissue specific antigens.

It has been proposed that NK cells play a regulatory function during human autoimmune diseases. Specifically, impaired NK cell activity was associated systemic onset of juvenile rheumatoid arthritis (JRA) and macrophage activation syndrome when compared to pauciarticular and polyarticular JRA (Grom et al., 2003; Villanueva et al., 2005). Consistently, within individuals suffering from type I diabetes after Coxsackie virus infection, the number of β cells in islets where increased when NK cells were present (Dotta et al., 2007; Lehuen et al., 2010). In animal models, NK cells may also mediate protective effects of CFA (complete Freund's adjuvant) in non-obese diabetic mice (Lee et al., 2008). Moreover, decreased NK cells promote the development of autoimmunity in C57BL/6 lpr mice (Takeda and Dennert, 1993). Our data indicate that infection with a higher virus dose may enhance the regulatory function of NK cells in order to prevent T cell mediated autoimmunity. NK cells are known to prevent autoimmunity during autoimmune encephalitis (Poggi and Zocchi, 2014). In patients, expansion of CD56^bright^ NK cells correlate with disease regression during therapy with daclizumab (Bielekova et al., 2006). Accordingly, NK cells inhibit pathologic disease progression during murine experimental autoimmune encephalitis (Zhang et al., 1997). Furthermore, blockade of the inhibitory NK cell receptor NKG2A alleviates the pathologic score by reducing autoreactive T cell immunity (Leavenworth et al., 2010). Considering our data, a high dose viral infection exhibits enhanced NK cell activation and up-regulates the activating NK cell receptor NKp46, alleviating autoreactive T cell immunity. Low dose infections however, may not result in regulatory NK cell functions thus allowing autoimmunity to happen.

NK cells are regulated by a variety of inhibitory and activating receptors. Previous studies indicate that NK cell regulatory ligand activity is clinically relevant for human infection. For instance, homozygous expression of the NK cell inhibitory receptor gene KIR2DL3 and its ligands, the HLA-C group 1 alleles, correlate positively with clearance of HCV (Khakoo et al., 2004). Conversely, the NK cell activating receptor gene KIR2DS3 is associated with elevated transaminases and persistence of seropositive HCV infection (Paladino et al., 2007). Based on our observations, it may be insightful to investigate the prevalence of CD8^+^ T cell mediated autoimmune diseases in patients with altered expression of NK regulatory receptors, such as KIR2DL3 and KIR2DS3. Furthermore, it may partially explain HLA driven predisposition to autoimmune disorders by altered CD8^+^ T cell functions in individuals with HLA haplotypes with variable activity toward inhibiting receptors on NK cells. Patient studies are warranted to investigate the scope of the mechanism we have uncovered in this report.

## Materials and Methods

### Mice, Virus, and Monitoring of Diabetes

C57BL/6J mice were obtained from The Jackson Laboratory. RIP-GP mice (C57Bl/6 background) have been previously described (Ohashi et al., 1991). NCR1^gfp/gfp^ mice have been previously described (Glasner et al., 2018). Wild-type LCMV (WE strain) was originally obtained from F. Lehmann-Grube (Weidt et al., 1995). To generate viral stocks, viruses were grown in L929 cells for 48 h and subsequently titrated as described previously (Battegay et al., 1991). Diabetes progression in RIP-GP mice was assessed by monitoring blood glucose levels following infection with 10^3^, 10^4^, or 10^5^ plaque-forming units (PFU) of wild-type LCMV-WE. A mouse was considered diabetic when its blood glucose reached 14 mM. We measured blood glucose using Contour glucometers (Bayer, Leverkusen). Viral titers in different organs were determined by plaque assays on MC57 cells, as previously described (Battegay et al., 1991). All mice were maintained under specific pathogen-free conditions at the Ontario Cancer Institute Animal Resource Center following institutional guidelines, or in accordance with LANUV under German laws for animal protection.

### Cytolytic T Lymphocyte Assay

Splenocytes from LCMV-infected mice were incubated for 5 h at 37°C in 96-well plates with EL-4 target cells previously loaded with LCMV-specific peptide and labeled with 400 μCi/ml ^51^Cr (Perkin Elmer). Eighty microliters of the culture supernatant was counted from each well using a Wallac Wizard counter (Perkin Elmer). Maximal release was induced by adding 100 μl of 1M HCl to target cells. Percent specific lysis was calculated as (c.p.m. sample release – c.p.m. spontaneous release)/(c.p.m. maximal release – c.p.m. spontaneous release) × 100. For NK cell-mediated killing, we followed the same protocol using YAC cells as targets. For NK cell – T cell assay, T cells were isolated from a naive mouse following stimulation with plate bound anti-CD3 plus soluble anti-CD28 (eBiosciences) antibodies in the presence of NK cells isolated from mice infected at the indicated doses. T cells were counted after 72 h.

### Flow Cytometry Analysis

For tetramer staining, splenocytes were stained using phycoerythrin-labeled MHC class I tetramer GP33/H-2D^b^, or NP396/H-2D^b^ for 15 min at 37°C, prior to the addition of an antibody specific for CD8 (BD PharMingen) for 20 min at 4°C, as previously described (Lang et al., 2013). For the analysis of IFN-γ, IL-2, and TNF-α intracellular expression, splenocytes were collected from mice 8 days after LCMV infection. The cells were restimulated *ex-vivo* in 96-well round-bottom plates (10^6^ cells/well) in Iscove's medium supplemented with 10% FCS and in presence of Brefeldin A (Pharmingen) and LCMV-specific MHC-I epitope peptides at a concentration of 10^−7^ M. After 5 h at 37°C, the cells were harvested, washed once with FACS buffer, and surface-stained with an allophycocyanin-Cy7-labeled anti-CD8 antibody. After washing, cells were stained for intracellular cytokines using the cytofix/cytoperm kit accordingly to manufacturer's instructions (Pharmingen). Cells were analyzed using a FACSCalibur equipped with Cellquest software (Becton Dickinson, San Jose, CA) or a FACS Canto equipped with DiVa software. The NKG2D tetramer was performed like previously described (Jamieson et al., 2002). NK cells were determined by anti-NK1.1 and anti-CD3 staining. All NK cell receptor antibodies and all NK cell receptor ligand antibodies were obtained from eBioscience.

### Immunohistochemistry

Freshly removed pancreata were immersed in phosphate-buffered saline (PBS) and snap-frozen in liquid nitrogen. For the staining of cell differentiation markers, frozen tissue sections (8 μm thick) were cut using a cryostat and stained as previously described (Lang et al., 2008) with primary rat monoclonal antibodies to CD8, CD4, MHC class I.

### ELISA

Sera IFN-α level was determined according to Invitrogen IFN alpha Mouse ELISA Kit's instruction.

### Statistical Analysis

Data are expressed as mean ± S.E.M. Statistical significant differences between two different groups were analyzed using student's *t*-test. Statistical difference between several groups was tested using one-way ANOVA with additional Bonferoni or Dunnett test. Statistically significant differences between groups in experiments involving more than one analysis time point were calculated using two-way ANOVA (repeated measurements). *p* < 0.05 were considered as statistically significant.

## Data Availability Statement

All datasets generated for this study are included in the article/Supplementary Material.

## Ethics Statement

The animal study was reviewed and approved by Ontario Cancer Institute Animal Resource Centre, Landesamt für Natur, Umwelt und Verbraucherschutz Nordrhein-Westfalen (LANUV).

## Author Contributions

PL and SC performed experiments and wrote the paper. HX, KL, LC, ED, MG, AP, VP, RW, TH, AE, and DM performed experiments. HC, AD, MR, DH, and TM provided reagents and discussed the data. PO initiated the study and wrote the paper.

### Conflict of Interest

The authors declare that the research was conducted in the absence of any commercial or financial relationships that could be construed as a potential conflict of interest.

## Supplementary Material

Supplementary Figure 1 Different infectious doses of LCMV affect diabetes induction but does not affect MHC-I upregulation in pancreatic islet cells. **(A)** RIP-GP mice were infected with 10^3^, 10^4^, or 10^5^ PFU of LCMV WE. Glycemia measurements are shown for individual mice following indicated doses of LCMV (percent incidence of diabetes for same experiment shown in Figure 1A for all doses). **(B)** C57Bl/6 mice were infected with 10^3^, 10^4^, or 10^5^ PFU of LCMV. MHC-I specific immunohistochemistry staining of snap frozen pancreas samples are shown. One representative of *n* = 3 is shown (scale bar = 50 μm).

Supplementary Figure 2 LCMV-NP specific CD8^+^ T cells responses are impaired after high dose infection compared to low dose infection. C57Bl/6 mice were infected with 10^3^ or 10^5^ PFU of LCMV-WE. 8 days post-infection, **(A)** np396-tetramer^+^CD8^+^ T cells were determined and **(B)** splenocytes were restimulated with the LCMV specific peptide np396 followed by measurement of intracellular IL-2 (left panel), TNF-α (middle panel), and IFN-γ (right panel) levels by flow cytometry (^***^indicates *p* < 0.001, *n* = 5).

Supplementary Figure 3 LCMV replication can be detected in the spleen but not in the pancreas after infection with 10^3^ or 10^5^ PFU LCMV. **(A–C)** C57Bl/6 mice were infected with 10^3^ or 10^5^ PFU of LCMV WE. **(A)** Virus titers in pancreas tissue were measured at the indicated time points following LCMV infection by plaque assay **(B,C)** Immunohistochemistry staining for the nucleoprotein of LCMV (clone: VL-4) was performed on sections obtained 3 days following LCMV infection in spleen **(B)**, and pancreas **(C)** sections (one representative image of *n* = 3 mice is shown, scale bar = 50 μm).

Supplementary Figure 4 4-1BB is upregulated in NK cells from high dose infected animals. **(A–D)** C57Bl/6 mice were infected with 10^3^ or 10^5^ PFU of LCMV WE. The expression of various surface markers and transcriptional factors indicated were determined in NK cells 2 days after infection (^*^indicates *p* < 0.05, ^**^indicates *p* < 0.01, ^***^indicates *p* < 0.001, *n* = 3–4).

Supplementary Figure 5 Similar early virus replication and normal IFN-I production in the presence or absence of NK cells. Control or NK cell depleted mice were infected with 10^3^ or 10^5^ PFU of LCMV WE. **(A)** At day 2 post-infection virus titer in spleen and pancreas tissues were measured (*n* = 4). **(B)** IFN-I level from sera at day 1 and day 2 post-infection was quantified (^*^indicates *p* < 0.05, ^***^indicates *p* < 0.001, *n* = 4).
