# Supplementary figures and images for: NK Cells Regulate CD8+ T Cell Mediated Autoimmunity
Source: Front Cell Infect Microbiol. 2020 Feb 13;10:36. doi: 10.3389/fcimb.2020.00036 (PMC7031256; doi:10.3389/fcimb.2020.00036)

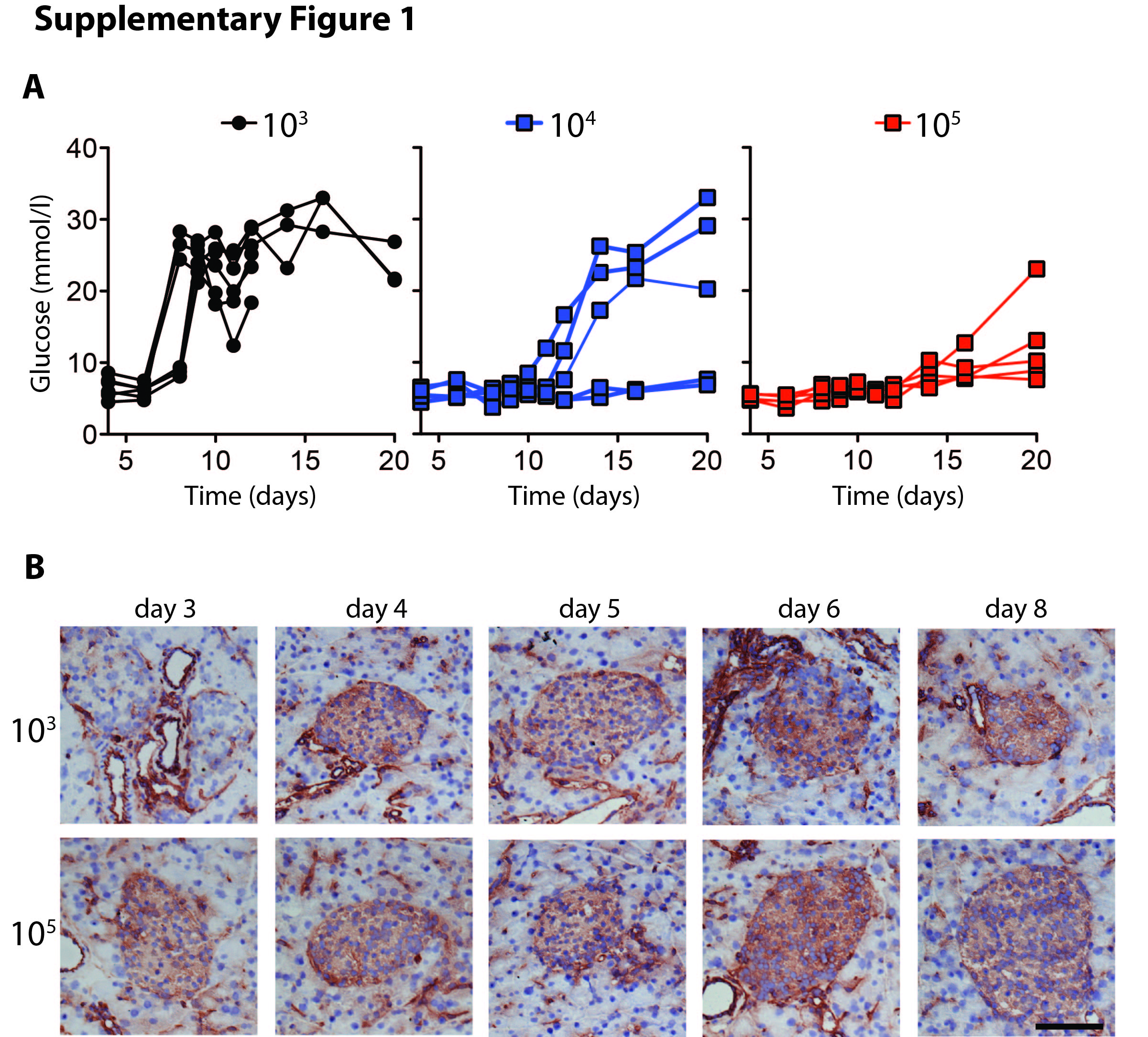

Supplement: Supplementary Figure 1 — Different infectious doses of LCMV affect diabetes induction but does not affect MHC-I upregulation in pancreatic islet cells. (A) RIP-GP mice were infected with 103, 104, or 105 PFU of LCMV WE. Glycemia measurements are shown for individual mice following indicated doses of LCMV (percent incidence of diabetes for same experiment shown in Figure 1A for all doses). (B) C57Bl/6 mice were infected with 103, 104, or 105 PFU of LCMV. MHC-I specific immunohistochemistry staining of snap frozen pancreas samples are shown. One representative of n = 3 is shown (scale bar = 50 μm). [file Image_1.JPEG]

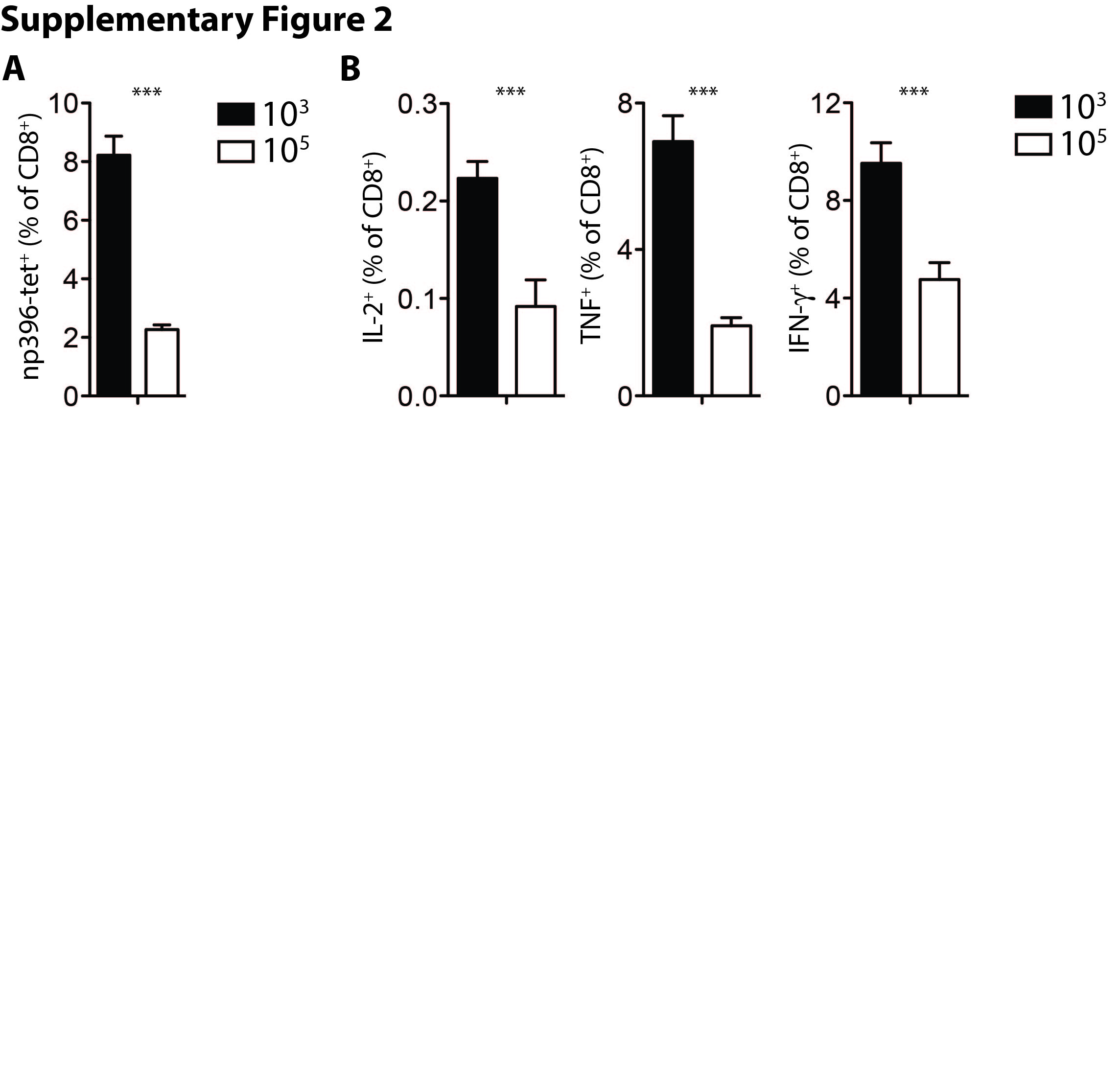

Supplement: Supplementary Figure 2 — LCMV-NP specific CD8+ T cells responses are impaired after high dose infection compared to low dose infection. C57Bl/6 mice were infected with 103 or 105 PFU of LCMV-WE. 8 days post-infection, (A) np396-tetramer+CD8+ T cells were determined and (B) splenocytes were restimulated with the LCMV specific peptide np396 followed by measurement of intracellular IL-2 (left panel), TNF-α (middle panel), and IFN-γ (right panel) levels by flow cytometry (***indicates p < 0.001, n = 5). [file Image_2.JPEG]

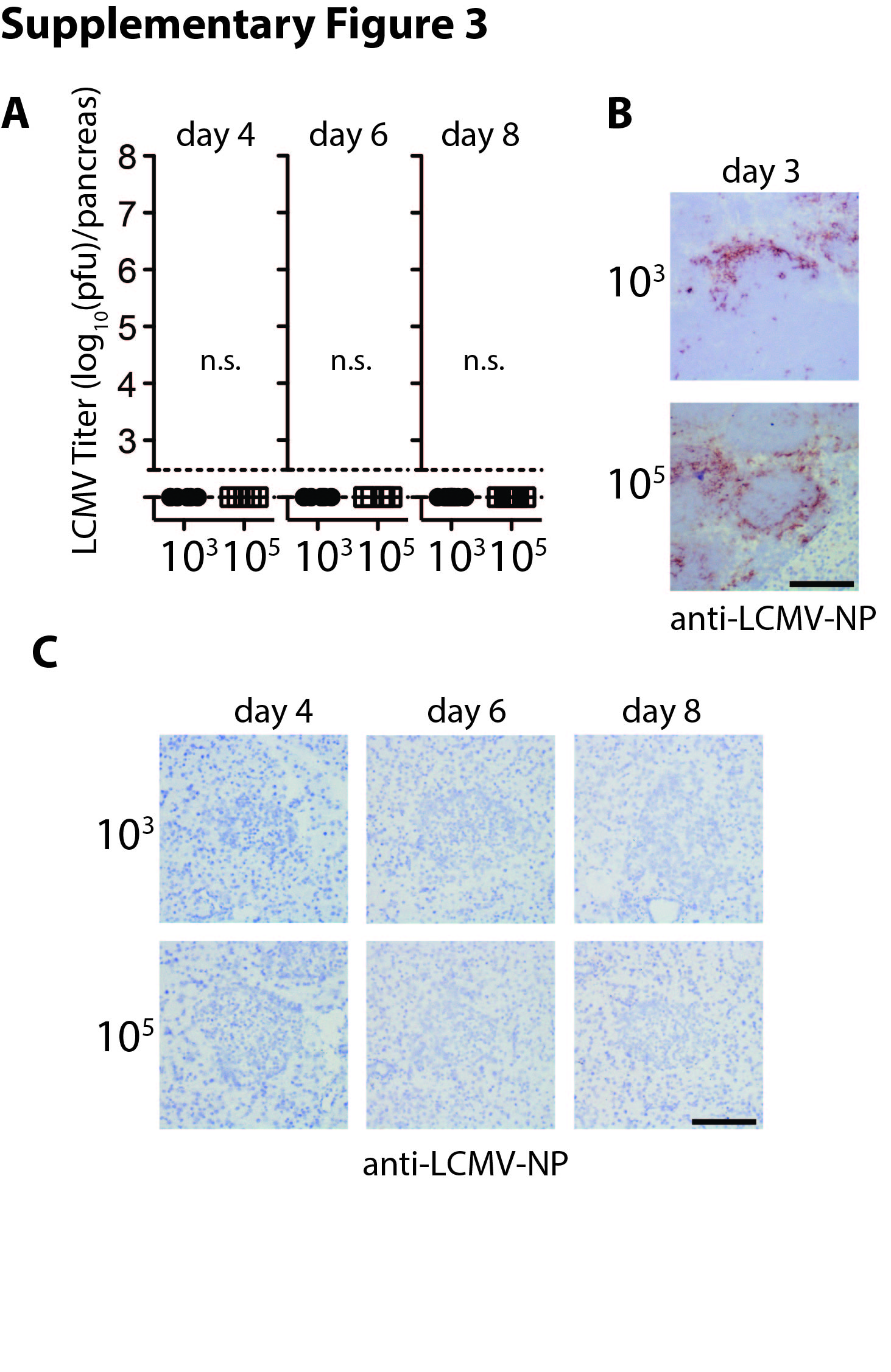

Supplement: Supplementary Figure 3 — LCMV replication can be detected in the spleen but not in the pancreas after infection with 103 or 105 PFU LCMV. (A–C) C57Bl/6 mice were infected with 103 or 105 PFU of LCMV WE. (A) Virus titers in pancreas tissue were measured at the indicated time points following LCMV infection by plaque assay (B,C) Immunohistochemistry staining for the nucleoprotein of LCMV (clone: VL-4) was performed on sections obtained 3 days following LCMV infection in spleen (B), and pancreas (C) sections (one representative image of n = 3 mice is shown, scale bar = 50 μm). [file Image_3.JPEG]

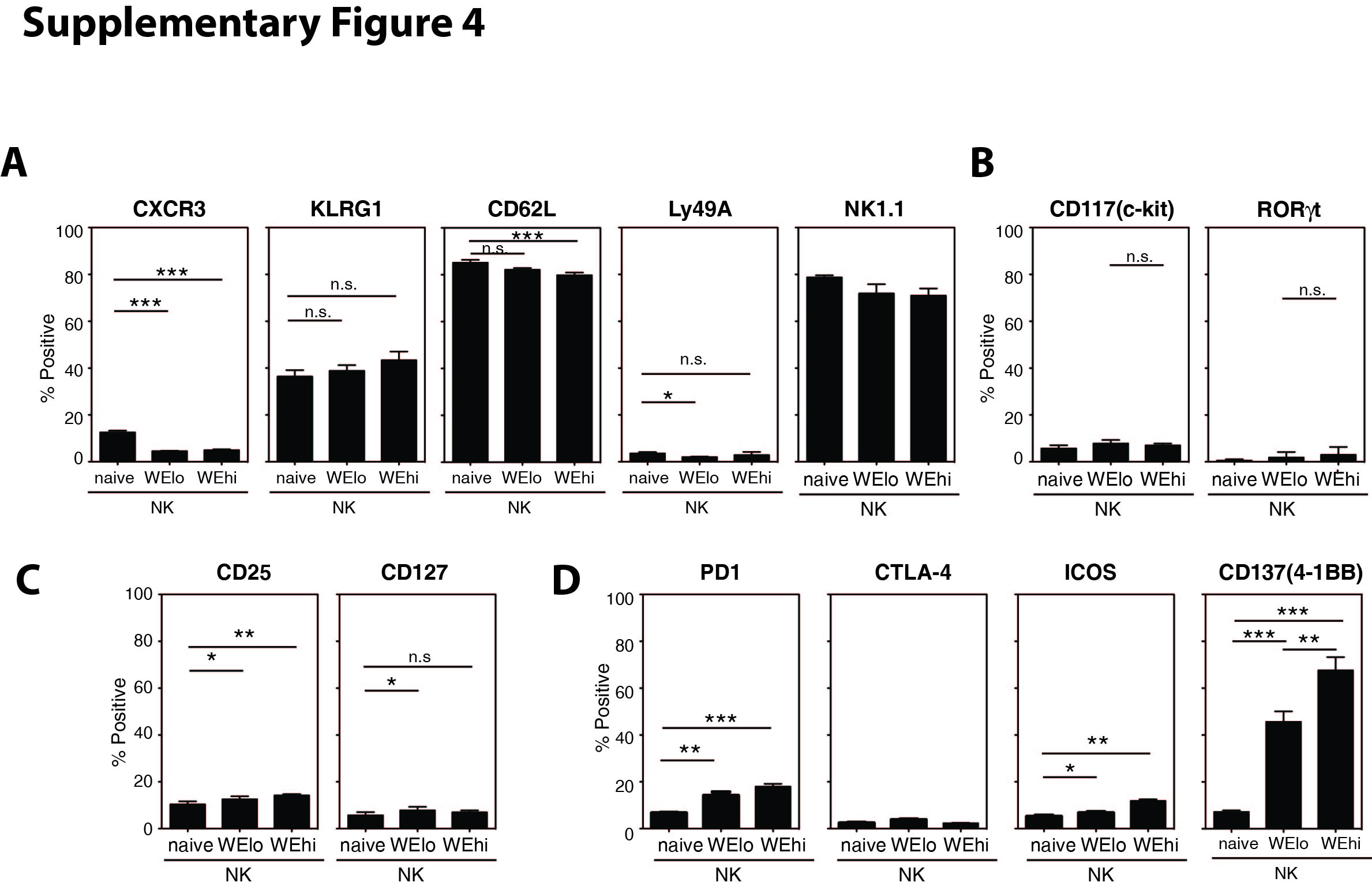

Supplement: Supplementary Figure 4 — 4-1BB is upregulated in NK cells from high dose infected animals. (A–D) C57Bl/6 mice were infected with 103 or 105 PFU of LCMV WE. The expression of various surface markers and transcriptional factors indicated were determined in NK cells 2 days after infection (*indicates p < 0.05, **indicates p < 0.01, ***indicates p < 0.001, n = 3–4). [file Image_4.JPEG]

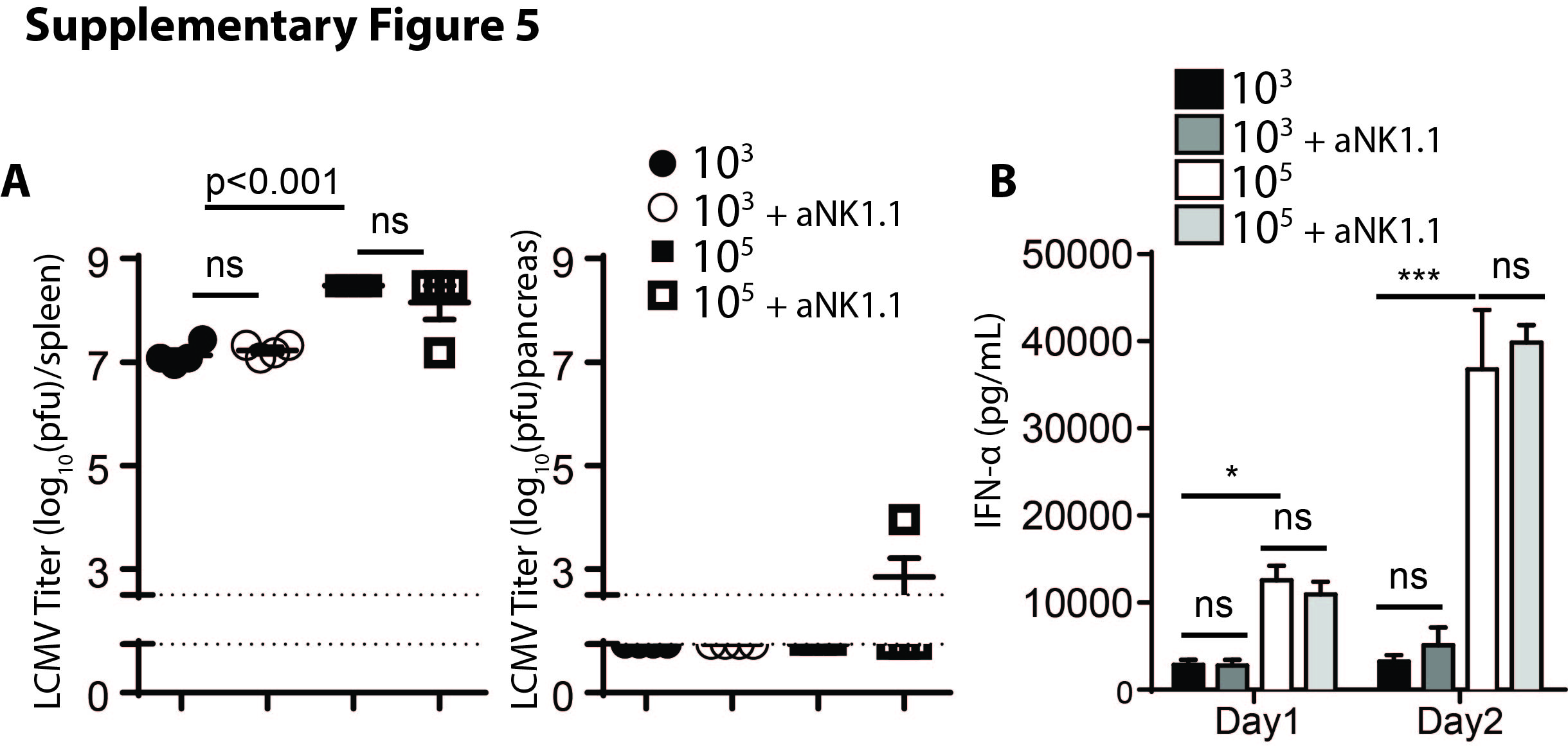

Supplement: Supplementary Figure 5 — Similar early virus replication and normal IFN-I production in the presence or absence of NK cells. Control or NK cell depleted mice were infected with 103 or 105 PFU of LCMV WE. (A) At day 2 post-infection virus titer in spleen and pancreas tissues were measured (n = 4). (B) IFN-I level from sera at day 1 and day 2 post-infection was quantified (*indicates p < 0.05, ***indicates p < 0.001, n = 4). [file Image_5.JPEG]
